# The major *TMEM106B* dementia risk allele affects TMEM106B protein levels, fibril formation, and myelin lipid homeostasis in the ageing human hippocampus

**DOI:** 10.1186/s13024-023-00650-3

**Published:** 2023-09-19

**Authors:** Jun Yup Lee, Dylan J Harney, Jonathan D Teo, John B Kwok, Greg T. Sutherland, Mark Larance, Anthony S Don

**Affiliations:** 1 Charles Perkins Centre, Camperdown, NSW 2006 Australia; 2 School of Medical Sciences, Camperdown, NSW 2006 Australia; 3 Brain and Mind Centre, The University of Sydney, Camperdown, NSW 2006 Australia

**Keywords:** TMEM106B, Proteomic, Lipidomic, Sphingolipid, Neurodegeneration, Ageing, Myelin, hippocampus

## Abstract

**Background:**

The risk for dementia increases exponentially from the seventh decade of life. Identifying and understanding the biochemical changes that sensitize the ageing brain to neurodegeneration will provide new opportunities for dementia prevention and treatment. This study aimed to determine how ageing and major genetic risk factors for dementia affect the hippocampal proteome and lipidome of neurologically-normal humans over the age of 65. The hippocampus was chosen as it is highly susceptible to atrophy with ageing and in several neurodegenerative diseases.

**Methods:**

Mass spectrometry-based proteomic and lipidomic analysis of CA1 hippocampus samples from 74 neurologically normal human donors, aged 66–104, was used in combination with multiple regression models and gene set enrichment analysis to identify age-dependent changes in the proteome and lipidome. ANOVA was used to test the effect of major dementia risk alleles in the *TMEM106B* and *APOE* genes on the hippocampal proteome and lipidome, adjusting for age, gender, and post-mortem interval. Fibrillar C-terminal TMEM106B fragments were isolated using sarkosyl fractionation and quantified by immunoblotting.

**Results:**

Forty proteins were associated with age at false discovery rate-corrected P < 0.05, including proteins that regulate cell adhesion, the cytoskeleton, amino acid and lipid metabolism, and ribosomal subunits. TMEM106B, a regulator of lysosomal and oligodendrocyte function, was regulated with greatest effect size. The increase in TMEM106B levels with ageing was specific to carriers of the rs1990622-A allele in the *TMEM106B* gene that increases risk for frontotemporal dementia, Alzheimer’s disease, Parkinson’s disease, and hippocampal sclerosis with ageing. Rs1990622-A was also associated with higher TMEM106B fibril content. Hippocampal lipids were not significantly affected by *APOE* genotype, however levels of myelin-enriched sulfatides and hexosylceramides were significantly lower, and polyunsaturated phospholipids were higher, in rs1990622-A carriers after controlling for *APOE* genotype.

**Conclusions:**

Our study demonstrates that TMEM106B protein abundance is increased with brain ageing in humans, establishes that dementia risk allele rs1990622-A predisposes to TMEM106B fibril formation in the hippocampus, and provides the first evidence that rs1990622-A affects brain lipid homeostasis, particularly myelin lipids. Our data suggests that *TMEM106B* is one of a growing list of major dementia risk genes that affect glial lipid metabolism.

**Supplementary Information:**

The online version contains supplementary material available at 10.1186/s13024-023-00650-3.

## Background

Ageing is the dominant, unifying risk factor for all major forms of dementia, and is thought to constitute the prodromal phase of neurodegeneration. An estimated 60–80% of dementia cases are Alzheimer’s disease (AD) [1], in which the hippocampus, a region of the brain that is essential for learning and memory, is affected early and heavily by neurofibrillary tangle (NFT) pathology and atrophy [2, 3]. Hippocampal pathology and degeneration are also characteristic of hippocampal sclerosis with ageing (HS-ageing), limbic-predominant age-related TDP-43 encephalopathy (LATE), frontotemporal dementia (FTD) and dementia with Lewy bodies [4–7]. Further to these dementia-related pathological changes, the hippocampus loses volume in the course of normal ageing [8], and this is associated with reduced verbal memory performance [3, 9].

Proteomic mass spectrometry permits the unbiased identification of cellular and biochemical changes in the brain as a function of ageing. This includes changes that cannot be detected using transcriptomics, such as molecular signatures arising from defective turnover and clearance pathways implicated in brain senescence [10, 11]. While there have been many studies characterizing transcriptomic changes in the ageing human brain [12, 13], few have investigated proteomic changes. Proteomic analyses of the rat, monkey, and human hippocampus reported changes to electron transport proteins [14–17], lysosomal proteins [14], redox control [15, 16], RNA splicing [14], and ribosomal stoichiometry [15] with increased age. However, the existing proteomic datasets for normal ageing in the human hippocampus are limited by small sample sizes [14, 17]. A recent, larger proteomic analysis of human frontal cortex demonstrated downregulation of mitochondrial and synaptic proteins in AD cases, whereas only synaptic proteins were inversely correlated with physiological ageing (ages 30 to 69) [18]. Another study established that mitochondrial, synaptic, and inflammatory protein networks are associated with cognitive trajectory [19], but did not investigate the effects of ageing on the brain proteome.

Although lipids make up > 50% of the dry weight of the brain [20], there have been very few studies on changes to the brain lipidome with ageing. Overall lipid content has been reported to decline with ageing in the human brain [21, 22], and this may be at least partly attributed to declining myelin content [23]. In contrast, myelin-enriched very long chain (24 carbon) ceramides and hexosylceramides increase with ageing in the mouse brain [21, 22].

Dementia risk doubles every 5 years after age 65 [1]. We therefore aimed to identify changes in the hippocampal proteome and lipidome of neurologically-normal humans over the age of 65 that sensitize to neurodegeneration. Protein networks associated with cytokine signaling and axon guidance were reduced with hippocampal age, whereas ribosomal, amino acid metabolism, and oxidative phosphorylation networks were increased. Levels of the lysosomal protein transmembrane protein 106B (TMEM106B) showed the greatest increase with ageing, and this effect was driven by the rs1990622-A allele in the *TMEM106B* gene, which is associated with cognitive decline in ageing and significantly increased risk for multiple forms of dementia. No lipids were significantly associated with age at death, however the *TMEM106B* rs1990622-A allele was associated with reduced myelin sphingolipid and higher polyunsaturated phospholipid content in the hippocampus. This is the first study to show an effect of this major dementia risk allele on brain lipid homeostasis.

## Methods

### Human tissue samples

Fresh-frozen hippocampus tissue samples (CA1 region) were provided by the New South Wales Brain Tissue Resource Centre (NSW BTRC) and Queensland Brain Bank. Samples were from donors with no neurological disorders at the time of death. Braak staging was performed as described [24], and showed an absence of NFT pathology (Braak 0) or NFT pathology restricted to the entorhinal cortex (Braak I/II) in the 66 cases for which this information was available [2]. Braak stage was not available for 8 donors. Age, post-mortem interval (PMI), *APOE* genotype, gender, *TMEM106B* rs1990622 genotype, cause of death, and Braak stage for each sample is provided in Supplementary Table 1. These cases were used for a previous targeted lipid analysis [25], however this paper reports new proteomic and lipidomic results and analyses. This work was approved by the University of Sydney Human Research Ethics Committee, approval #HREC2016/801.

Homogenates were prepared by placing 10–20 mg of frozen tissue into 500 µL of 20 mM Hepes pH 7.4, 10 mM KCl, EDTA-free cOmplete protease inhibitor cocktail (Roche), 1 mM dithiothreitol, and 3 mM β-glycerophosphate, and ultrasonicating for 5 min (30 s on/30 s off) at 4 °C in a Qsonica Q800R2 sonicating bath. Homogenates were cleared by centrifugation at 1000×g for 10 min at 4 °C, and the supernatant was stored at -80 °C in 100 µL aliquots. Total protein concentrations were determined using the Bradford assay (Bio-Rad).

### Proteomic sample preparation (NSW BTRC and QLD Brain Bank cohorts)

Crude homogenate (10 µg protein) was extracted with 100 µL 4% SDS, 100 mM NaCl, 20 mM NaPO_4_ (pH 6), 10 mM NaF, 10 mM tris(2-carboxyethyl)phosphine (TCEP), 10 mM N-ethylmaleimide (NEM), 10 mM sodium pyrophosphate, 2 mM sodium orthovanadate, 60 mM sodium β-glycerophosphate, and EDTA-free cOmplete protease inhibitor cocktail (Roche). The volume was made up to 150 µL using MilliQ water and samples were incubated for 10 min at 65 °C with shaking (500 rpm), then ultrasonicated for 10 min at 20 °C (15s on/15s off). Proteins were precipitated with chloroform/methanol/water in the ratio 1:4:1:3 (sample:methanol:chloroform:water) [26], dried down, and reconstituted in 30 µL of 8 M Urea in 0.1 M Tris-HCl (pH 8.0). Protein concentrations were measured by BCA assay (ThermoFisher Scientific), after which protein samples were diluted 8-fold in 0.1 M Tris-HCl (pH 8)/1 mM CaCl_2_ and digested for 16 h at 37 °C with 0.1 µg trypsin (#90,058, ThermoFisher). Digestion was stopped with the addition of trifluoroacetic acid to a final concentration of 1%, and the samples were centrifuged to pellet any undigested protein (18,000×g, 10 min, 20 °C). The supernatants were transferred to new tubes and subjected to solid-phase extraction [27].

### Preparation of sarksoyl-soluble and sarkosyl-insoluble protein fractions, and western blotting

Preparation of sarkosyl-insoluble and sarkosyl-soluble protein fractions was based on prior published protocols [28, 29]. In brief, 50 mg of frozen CA1 hippocampus tissue was homogenized in 16 vol (w/v) low salt buffer (50 mM HEPES pH 7.0, 250 mM sucrose, 1 mM EDTA) using 20–30 Eppendorf micropestle strokes. Homogenates were brought to 0.5 M NaCl and 1% sarkosyl (final tissue w/v 20) prior to sonicating at 30% intensity for 15 s with 5 s pulses, using a Qsonica Q800R2 sonicating bath. The homogenates were then incubated at RT for 30 min with shaking (650 rpm). Tissue debris was pelleted by centrifugation (870×g, 4 °C, 30 min) and discarded. The homogenates were then centrifuged at 180,000×g (4 °C, 30 min) to obtain the sarkosyl-soluble protein fraction. The pellet was resuspended in wash buffer (low salt buffer + 1% (w/v) sarkosyl and 0.5 M NaCl) and re-centrifuged (180,000×g, 4 °C, 30 min). This sarkosyl-insoluble protein pellet was resuspended in 50 µL of 8 M urea in 50 mM Tris HCl pH 8.5, 5 mM NaF, 2 mM Na_3_VO_4_, and EDTA-free cOmplete protease inhibitor cocktail for western blotting.

Proteins (7 µg/well) were resolved on Bolt™ 4–12% Bis-Tris Plus gels (ThermoFisher Scientific #NW04125BOX) and transferred to polyvinylidene fluoride membranes. Membranes were blocked for 1 h at RT with 5% skim milk in Tris-buffered saline containing 0.1% Tween-20 (TBST), then incubated overnight at 4 °C with primary antibodies; anti-C-terminus TMEM106B antibody (residues 204–253, Novus Biologicals, #NBP1-91311) diluted 1:300 in TBST with 2% skim milk or anti-N-terminus TMEM106B antibody (residues 1–50, Bethyl Laboratories, #A303-439 A) diluted 1:500 in 5% bovine serum albumin (Sigma Aldrich #A7906). Membranes were then incubated with horseradish peroxidase-conjugated anti-rabbit IgG-HRP diluted 1:5000 in TBST containing 5% skim milk for 2 h at RT. Membranes were imaged with ECL Ultra Western HRP Substrate (Millipore #WBULS0500) using a Bio-Rad ChemiDoc Touch. Bands were quantified by densitometry with Bio-Rad Image Lab software (v6.0.1).

### Proteomic analysis by nano-flow liquid chromatography-tandem mass spectrometry (LC-MS/MS)

Quantitative proteomics was conducted using data-independent acquisition on a Thermo Scientific Q-Exactive HF-X mass spectrometer coupled to an EASY-nLC system, using previously described chromatography and mass spectrometer parameters [30]. Raw data were searched with Spectronaut (version 12.0.20491.11.25225 Jocelyn) [30] using the Uniprot human database downloaded on 14th August 2019, yielding normalised abundances for 2615 proteins. Enzyme specificity was set to fully tryptic and semi-tryptic (cleavage C-terminal to Lys and Arg) with a maximum of two missed cleavages. Variable modifications consisted of deamidation of Asn and Gln, oxidation of Met, pyro-Glu (with peptide N-term Gln), and protein N-terminal acetylation. The iRT profiling workflow was used, and FDR was set to 1% based on a target decoy approach. All other settings were factory default. Proteins that were not detected in three or more samples were excluded from subsequent analyses, leaving 2091 proteins. Two samples were removed due to the absence of quality protein signals (< 800 proteins identified).

### Linkage disequilibrium analysis for rs1990622 single-nucleotide polymorphism (SNP)

Linkage disequilibrium between rs1990622 and all SNPs located ± 500,000 bp from the *TMEM106B* gene was determined using the LDproxy function from R package LDlinkR [31]. The squared correlation coefficients (r^2^) between rs1990622 and other SNPs was used as the measure of linkage strength, where r^2^ = 1 represents perfect linkage disequilibrium (coinheritance of SNPs). Analysis was performed using the 1000 Genomes Project database for Utah residents with Northern and Western European ancestry (CEU) and British in England and Scotland (GBR) populations [32].

### DNA extraction and TMEM106B rs1990622 genotyping

DNA was extracted from brain tissue using phenol-chloroform extraction [33]. Genotyping was performed using a TaqMan SNP genotyping assay for *TMEM106B* rs1990622 (ThermoFisher, Assay ID C_11171598_20, Catalog #4,351,379) as per manufacturer instructions. *APOE* genotypes for these cases were reported previously [25].

### Lipidomic analysis by LC-MS/MS

Lipids were extracted from hippocampal homogenates (~ 100 µg protein) using a one phase butanol-methanol (BUME) (1:1 v/v) procedure [34]. The following internal standards were added to each sample: 5 nmoles of d19:0/19:0 PC, 2 nmoles of d18:1/17:0 SM, d18:1/12:0 HexCer, 17:0/17:0 PS, 17:0/17:0 PE, 17:0/17:0/17:0 TG, 17:0/17:0 PG, 14:0/14:0/14:0/14:0 cardiolipin, 17:0 cholesteryl ester, 1 nmole of 17:0/17:0 PA, d18:1/15:0-d7 PI, cholesterol-d7, 0.5 nmoles of d18:1/17:0 ST, d18:1/17:0 ceramide, 17:1 LPE, 17:1 LPS, 17:0 LPC, 18:1-d7 monoacylglycerol, d18:1/15:0-d7 diacylglycerol, d18:1/12:0 Hex2Cer, and 0.2 nmoles of 17:1 sphingosine, 17:1 sphingosine 1-phosphate, d3-16:0 acylcarnitine, 17:0 LPA.

Lipids were detected using multiple reaction monitoring on a TSQ Altis triple quadrupole mass spectrometer with Vanquish HPLC (ThermoFisher Scientific). Precursor and product ion pairs are listed in Additional File 1. Lipids were resolved on a Waters Acquity UPLC CSH 2.1 × 100 mm C18 column (1.7 μm particle size) at a flow rate of 0.28 ml/min. Mobile phases were A: 10 mM ammonium formate, 0.1% formic acid, 60% acetonitrile and 40% water; B: 10 mM ammonium formate, 0.1% formic acid, 10% acetonitrile and 90% isopropanol. Run time was 25 min using a binary gradient starting at 20% B for 3 min, increasing to 45% B from 3 to 5.5 min, then to 65% B from 5.5 to 8 min, then to 85% B from 8 to 13 min, then to 100% B from 13 to 14 min. The gradient was held at 100% B from 14 to 20 min, then decreased to 20% B and held to 25 min. TraceFinder 4.1 (ThermoFisher) was used to integrate the peaks. The molar amount of each lipid was calculated with reference to its class-specific internal standard. As per Lipidomics Standards Initiative guidelines, each lipid was expressed as a molar % of total lipid quantified in that sample.

### Data analysis

All data and statistical analyses were performed using R. An a priori multiple regression power calculation was performed using the R package ‘pwr’ (pwr.f2.test) with 3 variables, a coefficient of determination (R^2^) of 0.3, Bonferonni corrected significance level under the assumption that 2000 proteins are quantified (α = 0.05/2000), and power set at 0.8, giving a conservative optimal sample size of 84.

Unless otherwise indicated, P values were adjusted for multiple comparisons using the Benjamini-Hochberg false discovery rate (FDR) correction at 5% (i.e. Q < 0.05 was considered significant). The normal distribution of residuals for all statistical models was assessed using the Anderson-Darling normality test. In cases where the test indicated non-normally distributed residuals, QQ plots and histograms were used to assess the degree of deviation from normality to decide whether variables should be natural log-transformed to fulfil the assumptions of normal distribution. Models that produced significant results but not fulfilling the assumptions of normal distribution with both linear and natural log-transformed continuous variables were considered false positives. One sample was identified as an outlier in both the proteomic and lipidomic datasets by hierarchical clustering and dendrogram analysis, using the hclust() function in R with average-linkage Euclidean distance measures between all samples. This sample was excluded from further analyses.

Associations between age and protein levels were tested by multiple regression with PMI and sex as co-variates (numerical variables natural log-transformed). The interaction between rs1990622 genotype and age in affecting TMEM106B protein levels was assessed using a multiple regression model, adjusting for PMI and sex. One-way ANOVA was used to test the effect of rs1990622 on TMEM106B protein levels, adjusting for age, PMI, and sex. Multiple regression models adjusted for age, PMI, and sex were used to test correlations between TMEM106B protein levels and the other 2090 proteins in the data set. Effects of *TMEM106B* rs1990622 and *APOE* genotype on the hippocampal lipidome and proteome were tested using one-way ANOVA, adjusting for age, PMI, and sex.

### Gene set enrichment analysis

Gene set enrichment analysis (GSEA) was performed using the clusterProfiler package [35]. The t-statistic value from each of the 2091 linear models assessing the association between age and the levels of each protein were used to rank proteins from the most positively to the most negatively correlated with age. The GSEA algorithm was then applied to this list using the GSEA() function, which checks whether a predefined set of proteins (e.g. mitochondrial oxidative phosphorylation) clusters at the top or bottom of the list to detect any coordinated upregulation or downregulation of protein sets with age. Protein sets used for the analyses were obtained from gene sets in the Molecular Signatures Database (v7.5.1 MsigDB, released January 2022). Gene sets containing less than 15 or more than 200 genes were excluded from the analyses. Statistical significance was determined based on permutation testing (10,000 permutations) using randomly generated gene lists, and significant enrichment was determined on the basis of Benjamini-Hochberg’s false discovery rate corrected P value of 0.05 (Q < 0.05). This workflow was also used for detecting coordinated changes in the proteome that are associated with TMEM106B protein abundance.

### Acquisition and processing of published proteomic and transcriptomic data

Raw files (.RAW) from the Johns Hopkins Ageing dorsolateral prefrontal cortex proteomic dataset (label-free quantification) were downloaded from Synapse (ID: syn20933797) [18]. Raw data was searched with DIA-NN 1.8 using the Uniprot human database downloaded on 7th May 2023. M/z ranges were set to 200–1800 for fragment ions and 300–1800 for precursors. Only tryptic peptides with a maximum of two missed cleavages (peptide length 7–30 and precursor charge 1–4) were considered in the search. N-terminal methionine excision was enabled, while methionine oxidation and N-terminal acetylation were set as variable modifications (up to five allowed per peptide). Cysteine carbidomethylation was assigned as a fixed modification. FDR for precursor identification was set to 1%. The search was run in three batches according to the run dates provided and the output files merged by protein IDs. Batch effects were removed from the combined output file by applying the Tunable Approach for Median Polish of Ratio (TAMPOR) algorithm using a hybrid of global internal standards (GIS) and protein signals in non-GIS samples [36]. Four outliers were detected and removed by hierarchical clustering after batch correction, using the hclust() function in R with average-linkage Euclidean distance measures between all samples.

Processed tandem mass tag labelling proteomic data from the Emory Alzheimer’s Disease Research Center (ADRC) cohort was accessed through the PRIDE repository (Project PXDO20296) [37], for both protein and peptide group data.

Processed mRNA microarray data from cognitively normal individuals of the National Institute of Mental Health (NIMH) and National Institute of Child Health and Human Development (NICHD) cohorts were acquired from the Gene Expression Omnibus under the accession number GSE30272 [38].

### Immunofluorescent staining for myelin

Paraffin-embedded, formalin-fixed Sect. (12 μm) from the hippocampal CA1 region of individuals aged 78 to 100 were acquired from the NSW Brain Tissue Resource Centre (University of Sydney Human Research Ethics Committee approval #HREC2016/801) and stained for myelin basic protein (MBP) [39]. Sections were heated at 60 °C for 30 min prior to dewaxing in xylene, then rehydrated in decreasing concentrations of ethanol and rinsed in distilled water. Antigen retrieval was performed by incubation in 10 mM sodium citrate buffer (pH 6.0) with 0.05% Tween-20 for 30 min at 98 °C, then formic acid for 8 min. After washing in phosphate buffered saline (PBS), the sections were blocked in 5% normal goat serum, 0.1% bovine serum albumin, 0.1% triton X-100 in PBS at RT for 1 h, then incubated overnight at 4 °C with mouse anti-MBP (R&D systems MAB42282) diluted 1:250 in blocking buffer. The sections were washed four times with PBST and incubated for 1 h at RT, in the dark, with anti-mouse Alexa Fluor 488 (Cell Signalling #4408, RRID: AB10694704) diluted 1:250 in blocking buffer. Sections were washed four times in PBS and counterstained with 1 µg/ml 4′,6-diamidino-2-phenylindole. Autofluorescence was eliminated using TrueBlack Plus autofluorescence quencher (Biotium, #23,007) according to the manufacturer’s protocol. Sections were coverslipped using ProLong Glass antifade (Life Technologies, P36980) and imaged on an Olympus VS-200 slide scanner. QuPath v.0.4.3 was used to calculate the mean fluorescence intensity and percentage area of MBP immunoreactivity in the CA1 region of the hippocampus.

## Results

### Ribosomal and respiratory proteins increase, and axon guidance proteins decrease, with age in the human hippocampus

**Fig. 1:**
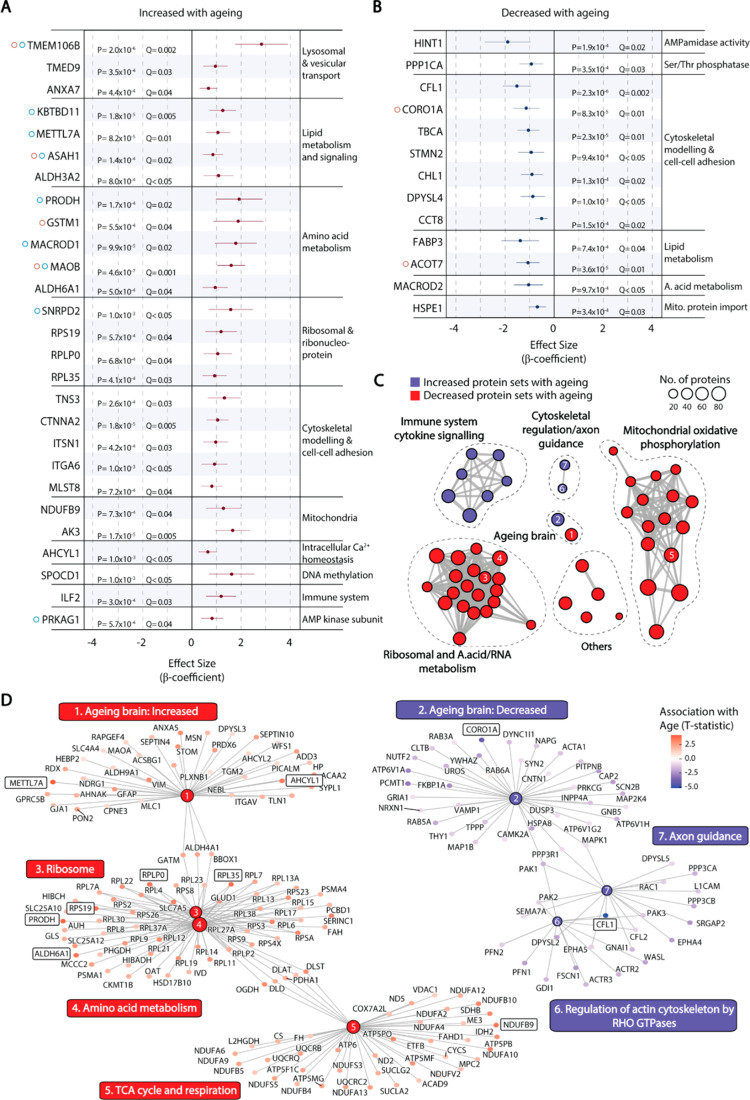
Proteins significantly correlated with age in human hippocampus. (**A-B**) Effect size estimates and 95% confidence intervals (horizontal bar) for proteins positively (**A**) or negatively (**B**) correlated with age at death (Q < 0.05) in linear regression models adjusted for PMI and sex. Proteins are grouped according to molecular functions from Uniprot. Proteins correlated with age in previously published ageing DLPFC datasets are indicated in red (Johns Hopkins Ageing cohort) and blue (Emory ADRC cohort) circles. (**C**) Protein set enrichment map of all significantly enriched categories from GSEA using curated protein sets from the molecular signatures database (MSIGDB, C2). Each node represents a significantly enriched protein set at Q < 0.05 that is increased (red) or decreased (blue) with age by the GSEA workflow. Protein sets with overlapping membership are connected by edges, where the thickness indicates the number of overlapping proteins. (**D**) Proteins that comprise specific nodes shown in (**C**). Nodes have been numbered to identify their position in the protein set enrichment map (**C**). Individual proteins that were significantly correlated with age at Q < 0.05 (**A,B**) are boxed

To identify proteins whose levels are regulated by age in the human hippocampus, proteomic analysis was performed on frozen tissue samples from the hippocampus CA1 region of 74 neurologically normal donors aged 66–104 (mean age at death 78 ± 8.4 years, 61% male) (Supplementary Table 1). Braak staging for NFT pathology was available for 66 samples, and all of these were Braak stage 0-II, indicating the absence of hippocampal NFT pathology that precedes neurodegeneration in AD [2]. After adjusting for false discovery rate (Q < 0.05) 27 proteins were positively correlated (Fig. 1A) and 13 negatively correlated (Fig. 1B) with age at death. These included components of ribosomes and mitochondria, as well as proteins involved in lysosome transport, lipid metabolism, amino acid metabolism, cytoskeletal modelling and cell-cell adhesion. Lysosomal transmembrane protein TMEM106B was regulated with the greatest effect size, increasing with age at death. Twelve of the 40 proteins that were correlated with age in our analysis of human hippocampus were also significantly correlated with age in either or both of two previously published proteomic datasets from dorsolateral prefrontal cortex of cognitively normal individuals: the Johns Hopkins Ageing cohort, age range 30–68 [18, 37], or the Emory ADRC Brain Bank, age range 45–96 [37] (Fig. 1A, B) (Supplementary Tables 2 and 3). With the exception of ASAH1, all of these were significant at P < 0.05, but not at Q < 0.05 when considering all proteins in the published datasets. ASAH1 was positively correlated with age in the Johns Hopkins Ageing cohort after adjusting for false discovery rate (β = 1.0, P = 3.5 × 10^− 6^, Q = 0.005).

Gene set enrichment analysis (GSEA) was applied to identify coordinated changes affecting biologically related groups of proteins. Proteins positively and negatively correlated with age in our dataset were significantly enriched in 42 and 10 curated gene sets in the Molecular Signatures Database (v7.5.1 MsigDB), respectively (Supplementary Table 4). Protein networks related to cytokine signaling, cytoskeletal regulation and axon guidance were decreased with hippocampal ageing, while protein networks involved in protein translation (ribosomes), amino acid and nucleotide metabolism, and oxidative phosphorylation were increased (Fig. 1C and D). Proteins that were positively associated with ageing in our dataset were significantly enriched for those whose gene expression increases with brain ageing, and downregulated proteins were enriched for those whose gene expression decreases with brain ageing in the human frontal cortex [13] (Fig. 1C and D). Importantly, several proteins that were significantly correlated with age at Q < 0.05 (Fig. 1A and B) were members of these significantly enriched protein sets (Fig. 1D). TMEM106B was not a member of any protein set that was significantly enriched in GSEA, suggesting that its upregulation does not form part of a coordinated response to ageing that can be categorized by predetermined gene sets.

### The correlation between TMEM106B and age is driven by the rs1990622-A dementia risk allele

**Fig. 2:**
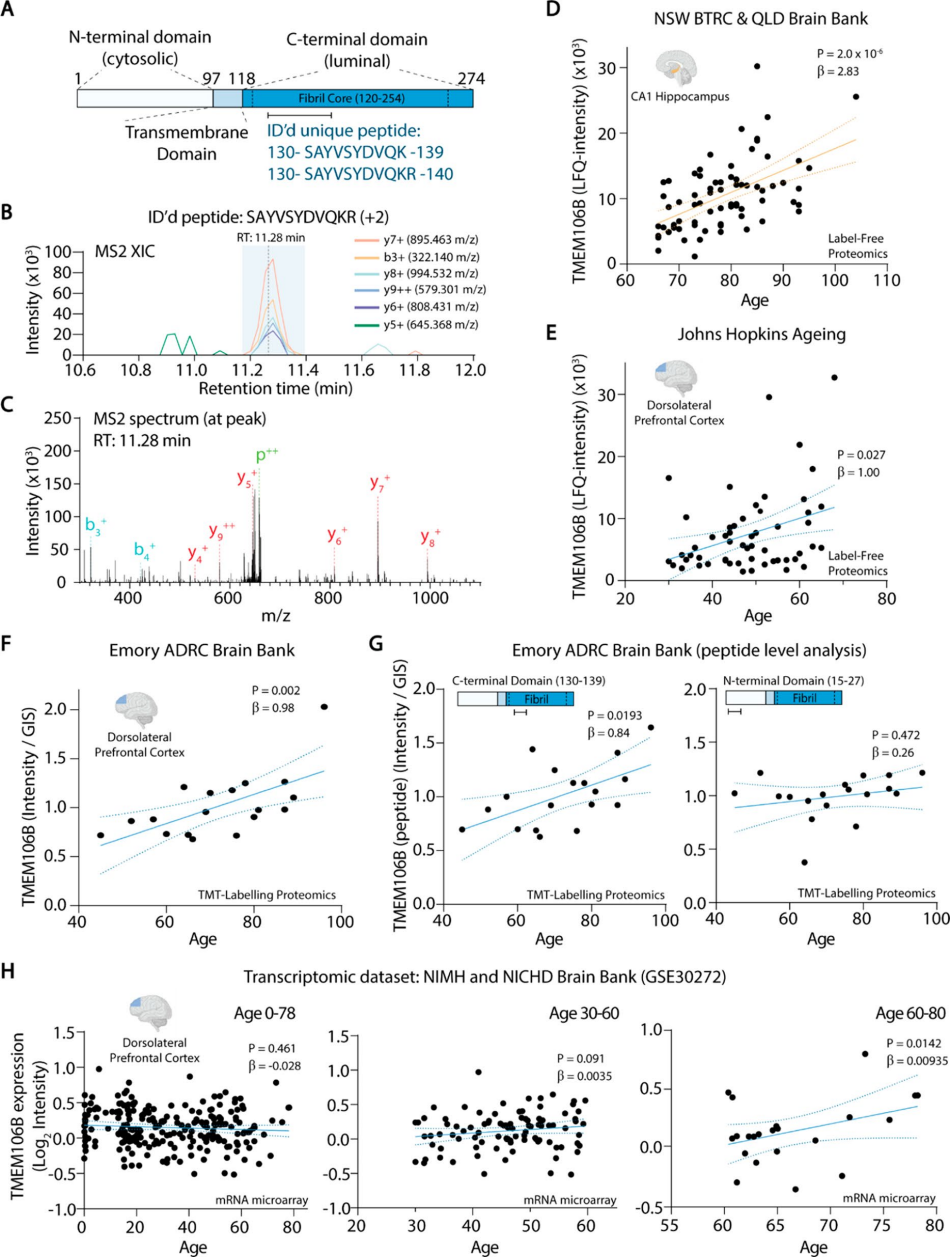
TMEM106B increases with age in the human brain. (**A**) Schematic of TMEM106B protein domains showing the unique peptide sequence used to identify and quantify TMEM106B protein levels in human hippocampus samples. (**B**) MS2 extracted ion chromatogram of the identified peptide (SAYVSYDVQKR) and (**C**) annotated MS2 fragmentation spectrum corresponding to the peak with retention time (RT) 11.28 min. (**D**) TMEM106B levels as a function of age in CA1 hippocampus samples from the NSW BTRC and QLD Brain Bank, (**E,F**) TMEM106B levels as a function of age in dorsolateral prefrontal cortex samples from the (**E**) Johns Hopkins Ageing (ages 30 to 68) and (**F**) Emory ADRC Brain Bank cohorts (ages 45 to 96). (**G**) Peptide-level associations of TMEM106B with age in the Emory ADRC Brain Bank cohort using peptides that map to the C-terminus (residues 130–139) (left) and the N-terminus (residues 15–27) (right). (**H**) *TMEM106B* mRNA levels as a function of age from GSE30272, using age ranges 0–78 (left), 30–60 (middle), and 60–80 (right)

The precise biochemical function of TMEM106B is unknown, however a dominantly inherited amino acid substitution (D252N) in the protein causes hypomyelinating leukodystrophy [40] and myelination defects are observed in TMEM106B knockout mice [41, 42]. At the cellular level, TMEM106B regulates lysosomal pH, size, transport and positioning [41–46]. Several recent studies have reported that a cleaved C-terminal fragment of TMEM106B forms amyloid fibrils with ageing and in neurodegenerative diseases [29, 47, 48]. In our dataset, TMEM106B was identified and quantified by peptides that map to its fibril forming C-terminal domain, spanning amino acids 120 to 254 (Fig. 2A-D). TMEM106B was quantified with the same C-terminal peptide in the published Johns Hopkins Ageing cohort [18], showing a similar increase in TMEM106B with age (Fig. 2E). In addition, TMEM106B increased with age in the Emory ADRC dataset [37], which used tandem mass tag proteomics (Fig. 2F). We restricted this analysis to Braak stages 0-IV, where there is minimal cortical presence of neurofibrillary tangles [2]. With the Emory ADRC dataset, we were able to analyse peptides mapping to the N- and C-termini of TMEM106B. The peptide mapping to the fibril-forming C-terminal domain (residues 130–139) increased with age, whereas a peptide mapping to the N-terminal domain (residues 15–27) did not (Fig. 2G). This suggests that increased TMEM106B with ageing may be attributed to accumulation of the C-terminal fibril-forming fragment of the protein.

No association between TMEM106B mRNA levels and age was observed in published microarray data from dorsolateral prefrontal cortex of cognitively normal individuals [GSE30272 [38]], when considering the full age range from 0 to 78 years (Fig. 2H). Narrowing the analysis to individuals aged 30–60 and 60–80 revealed a modest but statistically significant upregulation of *TMEM106B* gene expression after the age of 60 (Fig. 2G).

**Fig. 3:**
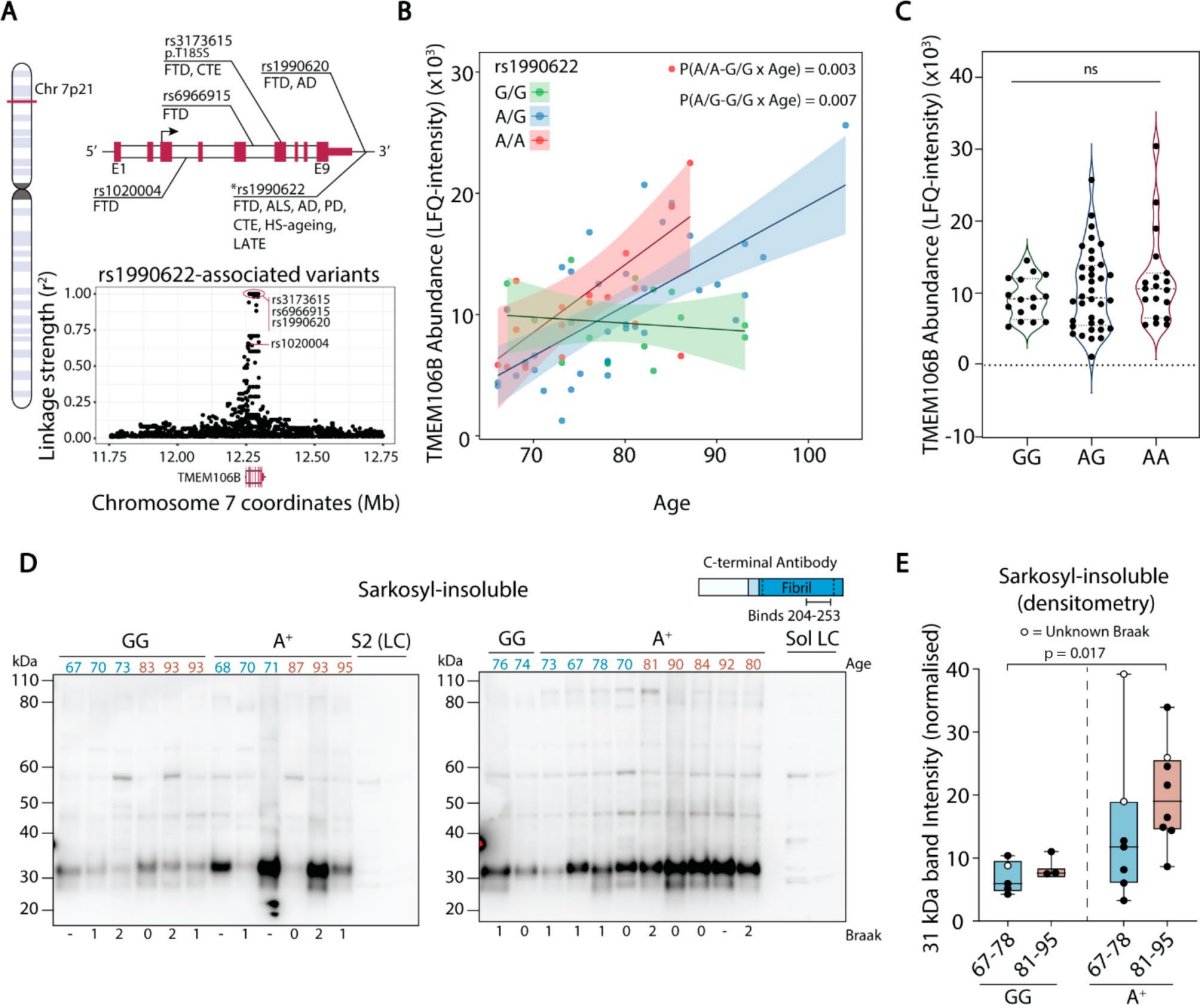
Hippocampal TMEM106B levels increase in carriers of the rs1990622-A risk allele. (**A**) (Top) Gene map of the *TMEM106B* locus (chromosome 7p21) with annotations of key *TMEM106B* SNP loci associated with neurodegenerative diseases (FTD: frontotemporal dementia; ALS: amyotrophic lateral sclerosis; AD: Alzheimer’s disease; PD: Parkinson’s disease; CTE: chronic traumatic encephalopathy; HS-ageing: hippocampal sclerosis with ageing; LATE: limbic-predominant age-related TDP-43 encephalopathy). Exons (E1-E9) are denoted in red, introns in white, and non-coding regions as a line. (Bottom) Linkage disequilibrium between rs1990622 and all SNPs located ± 500,000 bp from the *TMEM106B* gene as measured by the squared correlation coefficient (r^2^, where r^2^ = 1 is perfect linkage, depicted in red oval). Dementia risk-associated SNPs depicted in the gene map are labelled on the linkage plot. (**B**) TMEM106B protein levels as a function of age and rs1990622 genotype. P values refer to the interaction between rs1990622 genotype and age in multiple regression adjusted for PMI and sex. (**C**) TMEM106B levels as a function of rs1990622 genotype after adjusting for age, PMI, and sex (ANOVA, F = 2.7, P = 0.07). (**D**) Western blots for fibrillar TMEM106B in the sarkosyl-insoluble fraction using an anti-C-terminus antibody. Samples were from individuals aged 67–78 (blue) and 81–95 (red) that are homozygous for the rs1990622 G/G allele or carriers of the risk allele (A^+^). Braak stage of each individual is shown beneath the blot. Two sarkosyl-soluble samples were loaded onto the last two lanes of each blot to assess enrichment of TMEM106B fibrils (Sol LC). (**E**) Densitometry quantification of the 31 kDa bands observed in (**D**)

A SNP in the 3’ untranslated region of *TMEM106B* (rs1990622-A) is associated with increased risk for AD, FTD, Parkinson’s disease, HS-ageing, and LATE [4, 43, 49, 50], and is in perfect linkage disequilibrium with other SNPs in the *TMEM106B* gene that modify dementia risk (Fig. 3A). We obtained rs1990622 genotype frequencies of 49% G and 51% A (26% A/A, 51% A/G, 23% G/G) for our cohort, in agreement with reported values [50]. The increase in hippocampal TMEM106B levels with age was specific to carriers of the rs1990622-A risk allele and not observed in homozygous carriers of the protective G allele (Fig. 3B). When the effect of age was regressed out by performing an ANOVA adjusted for age, sex, and PMI, TMEM106B protein levels were no longer significantly affected by rs1990622 genotype (Fig. 3C).

### Accumulation of TMEM106B fibrils in the hippocampus of rs1990622-A allele carriers

We next used a sarkosyl fractionation protocol on a subset of our sample cohort to determine if higher levels of TMEM106B in carriers of the rs1990622-A allele could be attributed to accumulation of the sarkosyl-insoluble fibrillar C-terminal fragment. Immunoblotting the sarkosyl-insoluble fraction using an antibody that binds to the C-terminal region of TMEM106B (residues 204 to 253) revealed a prominent band at 31–33 kDa that is not observed in the sarkosyl-soluble fraction (Fig. 3D). Recent publications have established that this band corresponds to TMEM106B fibrils [29, 47, 51]. Compared to the protective rs1990622-G/G genotype, rs1990622-A allele carriers (A^+^) showed significant accumulation of sarkosyl-insoluble TMEM106B in the hippocampus (Welch’s T-test, P = 0.0026) (Fig. 3D). Stratifying the samples into younger (age 67–78, median 70.5) and older (age 81–95, median 90) age groups, we noted that sarkosyl-insoluble TMEM106B trended higher in the older rs1990622-A^+^ group (Fig. 3E). However, this was not statistically significant by 2-way ANOVA [Effect of genotype (F = 21.6, P = 0.018); age (F = 8.5, P = 0.12); age-genotype interaction (F = 0.93, P = 0.60)], due to variability attributed to high TMEM106B fibril levels in a single case with unknown Braak stage in the younger age group. Considering only the samples with confirmed Braak stage 0-II (i.e. absence of hippocampal neurofibrillary tangle pathology), TMEM106B fibril abundance was significantly affected by both rs1990622 genotype and age [Effect of genotype (F = 14.3, P = 0.043); age (F = 23.3, P = 0.013); age-genotype interaction (F = 4.2, P = 0.25)].

We were unable to quantify full-length TMEM106B in our human hippocampus samples, as neither the C-terminal antibody nor an antibody to the N-terminus produced a clear band at the expected size for full-length TMEM106B (43–50 kDa) [46, 51–53] in immunoblots of the sarkosyl-soluble fraction. Using a different C-terminal antibody, Vicente et al. were also able to detect sarkosyl-insoluble fibrillar TMEM106B but not sarkosyl-soluble TMEM106B [51].

### TMEM106B levels are associated with lipid metabolic and myelination proteins

**Fig. 4:**
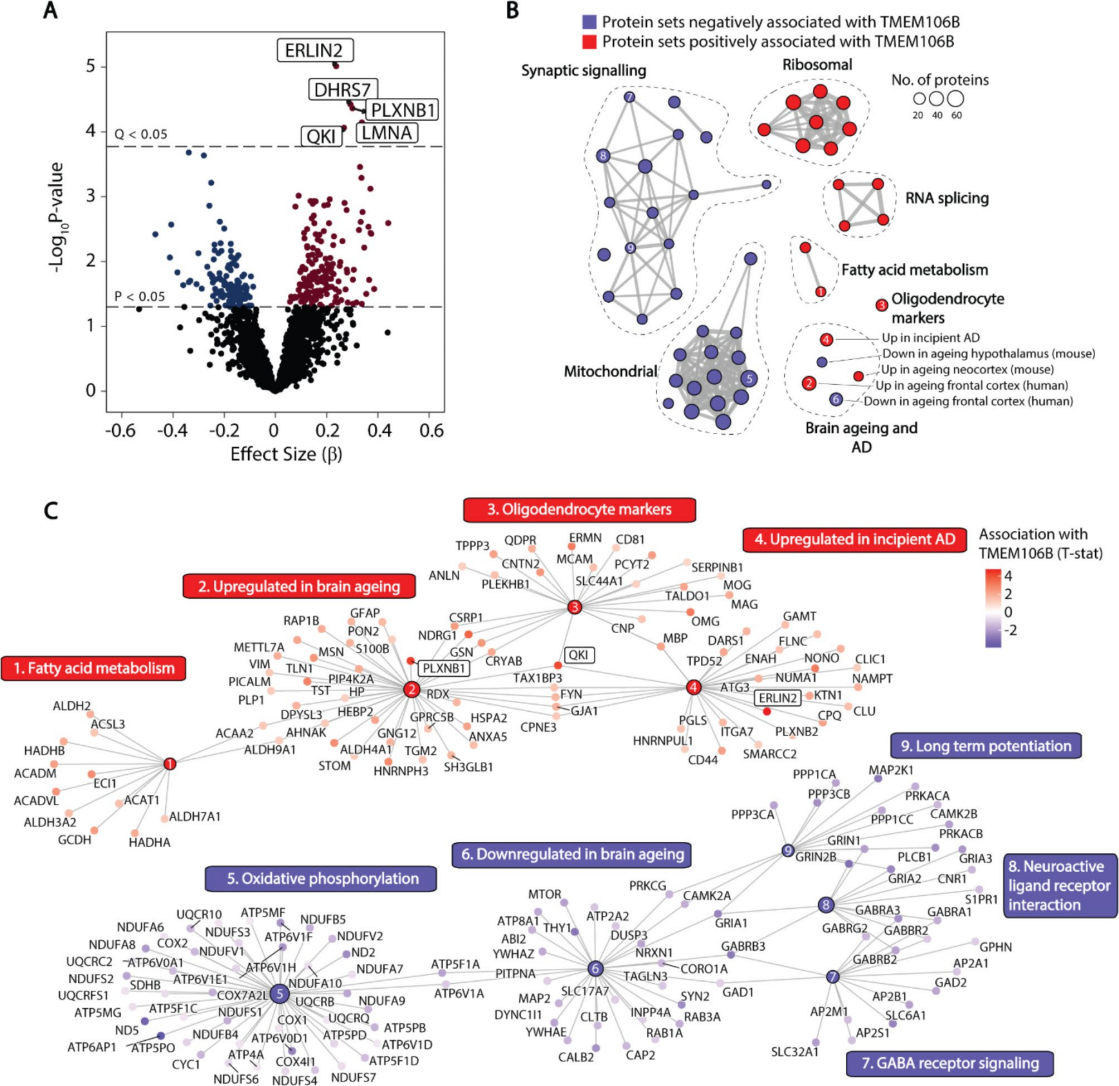
TMEM106B levels are correlated with proteins involved in myelination and brain ageing. (**A**) Proteins correlated with TMEM106B in multiple regression adjusted for PMI, sex, and age. Proteins significant at Q < 0.05 are boxed and labelled. (**B**) Protein set enrichment map of selected significantly enriched categories from GSEA using curated protein sets from the molecular signatures database (MSIGDB, C2). Each node represents a significantly enriched protein set at Q < 0.05 that is negatively (blue) or positively (red) associated with TMEM106B levels. Protein sets with overlapping membership are connected by edges, where the thickness indicates the number of overlapping proteins. (**C**) Protein membership of specific nodes shown in (**B**). Nodes have been numbered to identify their position in the protein set enrichment map (**B**). Proteins that were significantly correlated with TMEM106B levels at Q < 0.05 are boxed

To gain further insight into the function of TMEM106B and the implications of higher TMEM106B levels in the hippocampus, we identified proteins whose levels were correlated with TMEM106B after adjusting for age (Fig. 4A). Five proteins were positively correlated with TMEM106B at Q < 0.05: ER lipid raft associated 2 (ERLIN2), an endoplasmic reticulum protein that regulates lipid metabolism [54] and cell cycle progression [55]; dehydrogenase/reductase 7 (DHRS7), a member of the short-chain dehydrogenase/reductase family involved in the metabolism of retinoids and sterols [56]; the RNA-binding protein quaking I (QKI), a master regulator of oligodendrocyte differentiation, lipid biosynthesis, and myelination [57, 58]; the semaphorin receptor plexin B1 (PLXNB1), which is involved in synapse formation and axonal guidance [59, 60]; and nuclear envelope protein lamin A/C (LMNA). No proteins were regulated by rs1990622 genotype at Q < 0.05 (Supplementary Table 5). Considering only Braak 0-IV cases, no proteins were significantly correlated at Q < 0.05 with TMEM106B in the published prefrontal cortex Emory ADRC [37] or Johns Hopkins Ageing [18] cohort data after adjusting for age, sex, and PMI. ERLIN2 was correlated with TMEM106B at P < 0.05 in the Emory ADRC study, after restricting the analysis to ages 60 and above to match our analysis (β = 0.14, P = 0.031).

GSEA demonstrated that TMEM106B levels are negatively associated with synaptic signaling and oxidative phosphorylation protein networks, and positively associated with ribosomal, RNA splicing, fatty acid metabolism, and oligodendrocyte protein networks (Fig. 4B and C, and Supplementary Table 6). Even after adjusting for age, TMEM106B levels were positively correlated with protein sets whose gene expression increases with age in the human frontal cortex [13] and mouse neocortex [61], and negatively correlated with proteins sets whose gene expression decreases in the ageing human frontal cortex [13] and mouse hypothalamus [62] (Fig. 4C). Interestingly, TMEM106B levels were also positively associated with proteins whose gene expression is increased in the hippocampus during incipient AD [63].

### Myelin lipid content is decreased in carriers of the rs1990622-A dementia risk allele

**Fig. 5:**
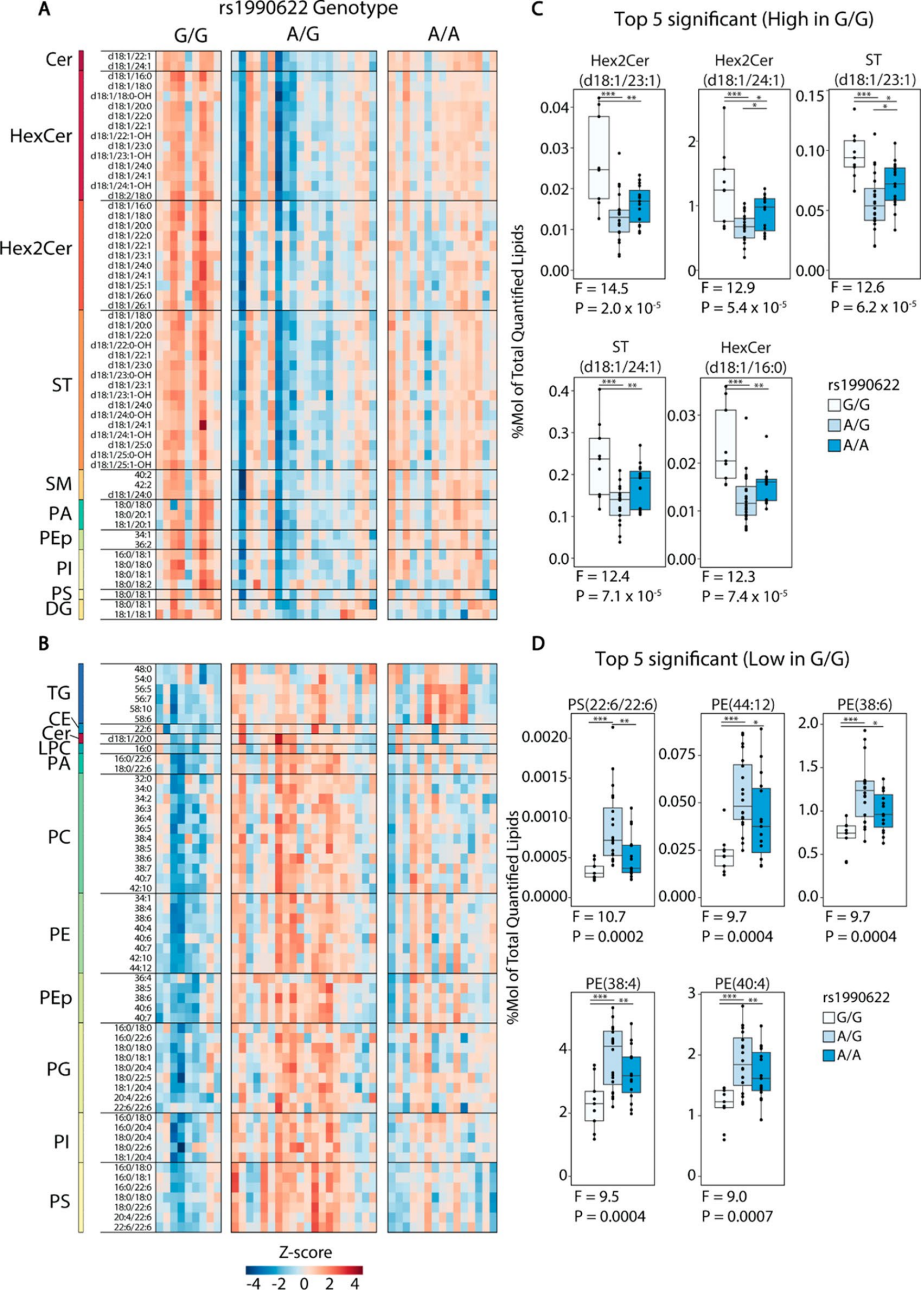
*TMEM106B* rs1990622 genotype affects the hippocampal lipidome. (**A-B**) Heatmap of lipids that were significantly affected by rs1990622 genotype (N_G/G_=9, N_A/G_=20, N_A/A_=15), in individuals with *APOE ε3/ε3* genotype. Each row represents a lipid that was significantly more abundant (**A**) or less abundant (**B**) in rs1990622-G/G individuals by ANOVA at Q < 0.05. (**C-D**) Top five lipid species that are more abundant (**C**) and less abundant (**D**) in rs1990622-G/G individuals ranked by P value. Lipids values are expressed as a molar % of total lipid. Cer: ceramide; DG: diacylglycerol; HexCer: hexosylceramide; Hex2Cer: dihexosylceramide; LPA: lysophosphatidic acid; LPC: lysophosphatidylcholine; PA: phosphatidic acid; PC: phosphatidylcholine; PE: phosphatidylethanolamine; PEp: phosphatidylethanolamine plasmalogen; PG: phosphatidylglycerol; PI: phosphatidylinositol; PS: phosphatidylserine; SM: sphingomyelin; ST: sulfatide; TG: triglyceride; CE: cholesterol ester

Given that (i) many of the most important gene variants affecting dementia risk regulate lipid transport and catabolism [64], (ii) structural modelling has proposed a lipid binding function for TMEM106B [65], and (iii) TMEM106B protein levels were correlated with proteins regulating fatty acid and lipid metabolism, we used lipidomic analysis to determine how dementia risk allele rs1990622-A affects lipid composition in the hippocampus of cognitively normal humans. A total of 234 phospholipids, sphingolipids, and neutral lipids were quantified by LC-MS/MS. As variants in *APOE*, which encodes a lipid transport protein, are the most significant determinant of genetic risk for dementia [64], we first determined whether lipid levels were significantly affected by *APOE* genotype. In one-way ANOVA comparing 46 cases with a risk-neutral *APOE* ε3/ε3 genotype, 12 with a protective ε3/ε2 or ε2/ε2 genotype (ε2 carriers), and 12 with a risk-increasing ε3/ε4 genotype, no lipids were significantly regulated (Q < 0.05) at the level of individual lipid species or lipid class totals (Supplementary Tables 7 and 8). Similarly, no lipids were regulated by rs1990622 genotype at Q < 0.05 in the full sample set (Supplementary Table 9). However, when the effect of rs1990622 was tested in *APOE* ε3/ε3 samples alone, 57 lipids were more abundant and 57 less abundant in carriers of the protective rs1990622-G/G genotype compared to rs1990622-A allele carriers (Fig. 5A-D, and Supplementary Table 10).

**Fig. 6:**
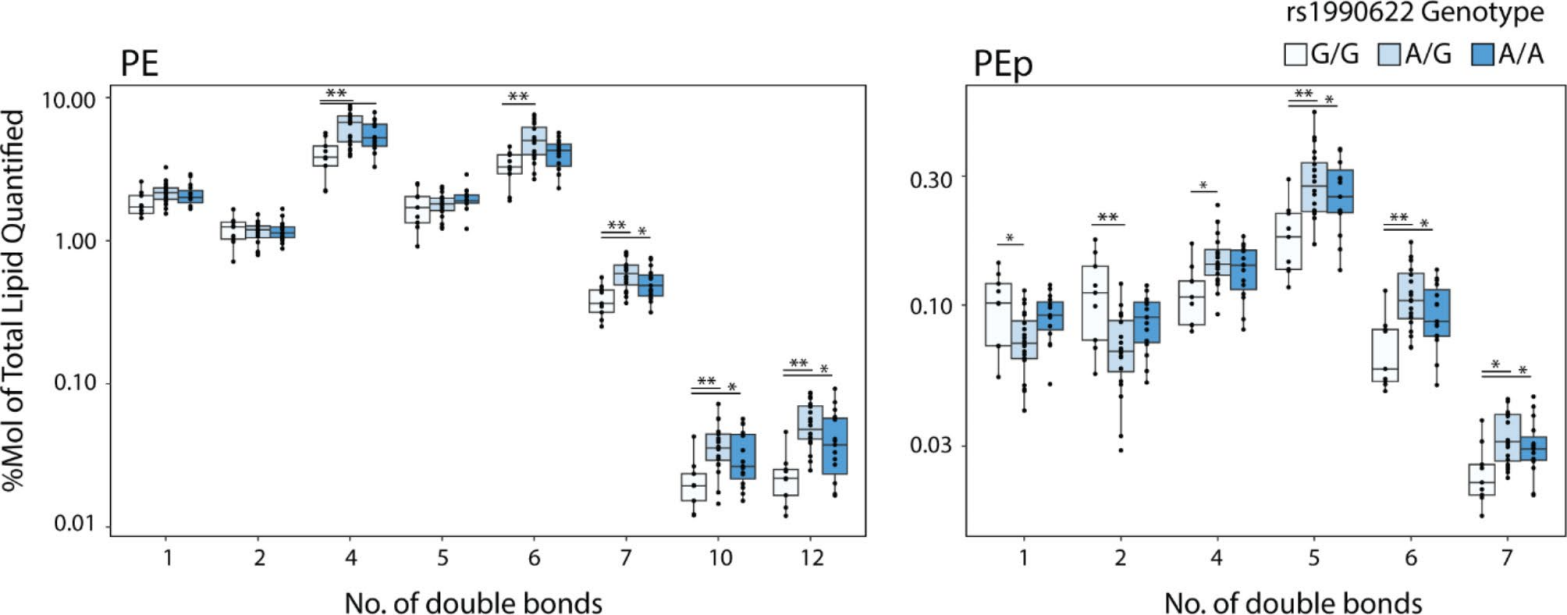
Rs1990622 affects the degree of unsaturation in phosphatidylethanolamine (PE) and PE plasmalogen (PEp). PE and PEp levels as a function of the number of double bonds in the acyl chains, grouped by rs1990622 genotype (*APOE* ε3/ε3 genotype only; N_G/G_=9, N_A/G_=20, N_A/A_=15). Statistical significance was determined by ANOVA with Tukey’s post-hoc test: *P < 0.05, **P < 0.01, ***P < 0.001

Of the 57 lipids that were higher in G/G individuals, 45 were sphingolipids, specifically hexosylceramides (HexCer), dihexosylceramides (Hex2Cer), sulfatides (ST), sphingomyelins (SM), and ceramides (Cer). Over 99% of HexCer in the brain is galactosylceramide [66], which makes up 20–25% of myelin lipids [20]. Together with ST (4–5% of myelin lipid), galactosylceramide is essential for myelin stability, and these lipid classes are unique to myelin in the CNS [67]. All but two of the lipids that were lower in rs1990622-G/G genotype individuals consisted of phospholipids and triglycerides (TG), particularly those with polyunsaturated fatty acid chains (Fig. 5B and D). The effect of rs1990622 on saturated versus polyunsaturated lipids was particularly apparent for phosphatidylethanolamine plasmalogens (PEp): PEp species with 1 or 2 double bonds were higher in people with a rs1990622-G/G genotype, whereas those with 4 or more double bonds were decreased (Fig. 6).

**Fig. 7:**
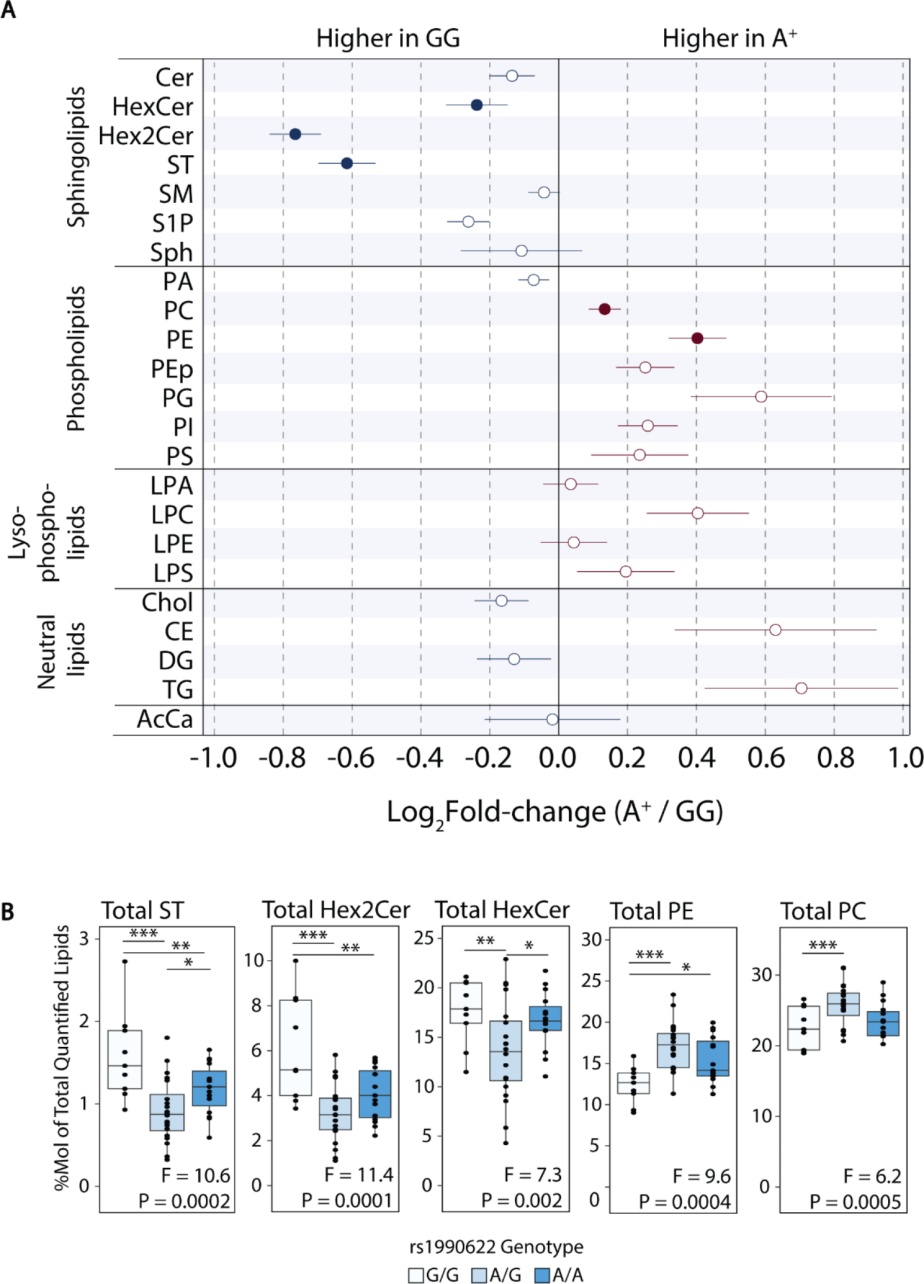
Higher ST and Hex2Cer, and lower PE, in carriers of the protective rs1990622-G/G genotype. (**A**) Summary of changes in lipid class totals in rs1990622-A allele carriers (denoted as A^+^) relative to rs1990622-G/G homozygotes (*APOE* ε3/ε3 genotype only). Filled circles indicate lipid classes that differed significantly between the three rs1990622 genotypes (G/G, A/G, and A/A) by ANOVA, at Q < 0.05 (*APOE* ε3/ε3 genotype only; N_G/G_=9, N_A/G_=20, N_A/A_=15). (**B**) Lipid class totals as a function of rs1990622 genotype for the five lipids significant by ANOVA. F and P values are shown at the bottom of each plot and results of Tukey’s post-hoc comparisons are shown above (* P < 0.05, ** P < 0.01, *** P < 0.001). Cer: ceramide; HexCer: hexosylceramide; Hex2Cer: dihexosylceramide; ST: sulfatide; SM: sphingomyelin; S1P: sphingosine 1-phosphate; Sph: sphingosine; PA: phosphatidic acid; PC: phosphatidylcholine; PE: phosphatidylethanolamine; PEp: phosphatidylethanolamine plasmalogen; PG: phosphatidylglycerol; PI: phosphatidylinositol; PS: phosphatidylserine; LPA: lysophosphatidic acid; LPC: lysophosphatidylcholine; LPE: lysophosphatidylethanolamine; LPS: lysophosphatidylserine; Chol: cholesterol; CE: cholesteryl ester; DG: diacylglycerol; TG: triacylglycerol; AcCa: acylcarnitine

At the lipid class level (sum of individual lipid species), Hex2Cer and ST were higher, and PE was lower, in the rs1990622-G/G compared to both A/G and A/A genotype groups (Fig. 7A and B, Supplementary Table 11). Total HexCer was higher, and PC was lower, in the G/G compared to the A/G genotype group, but these lipid class totals did not differ between the G/G and A/A genotype groups. Other sphingolipid, phospholipid, and neutral lipid class totals were unaffected by rs1990622 genotype at Q < 0.05. TMEM106B protein levels were not significantly correlated with any lipids or lipid class totals, and no individual lipids or lipid class totals were significantly correlated with age (Supplementary Tables 12 and 13).

**Fig. 8:**
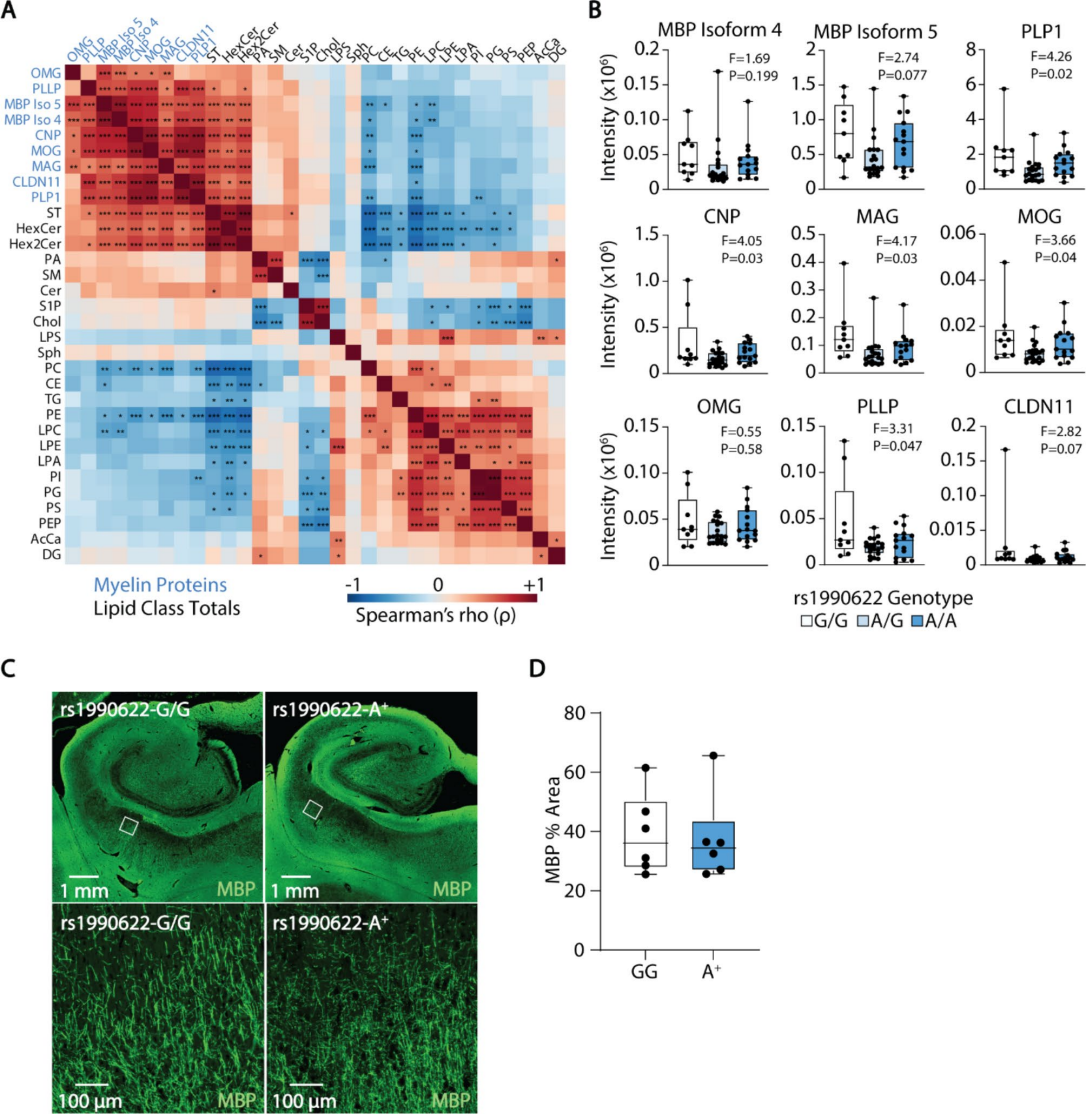
Rs1990622 genotype does not significantly alter myelin protein levels. (**A**) Spearman’s correlation matrix between myelin proteins (blue labels) and lipid class totals (black labels). Asterisks show Bonferonni-corrected P values (* P < 0.05, ** P < 0.01, *** P < 0.001). (**B**) Myelin sheath proteins as a function of rs1990622 genotype. ANOVA F and P values are shown above each plot (none significant at P < 0.05 after correcting for false discovery rate). (**C**) Representative MBP immunostaining, and (**D**) % area of MBP staining in the CA1 region of the hippocampus of individuals that are homozygous for the rs1990622-G/G allele (n = 6) and carriers of the risk allele (rs1990622-A^+^, n = 6), aged 78–100. Lower images in (**C**) show high magnification images of boxed region (white box). CLDN11: Claudin-11; CNP: 2’,3’-Cyclic nucleotide 3’-phosphodiesterase; MAG: Myelin-associated glycoprotein; MBP: Myelin basic protein; MOG: Myelin-oligodendrocyte glycoprotein; OMG: Oligodendrocyte-myelin glycoprotein; PLLP: Plasmolipin; PLP1: proteolipid protein 1

As expected, the abundance of myelin-enriched sphingolipids (ST, HexCer, Hex2Cer) was positively correlated with myelin proteins across our sample cohort (Fig. 8A). Levels of the myelin proteins PLP1, CNP, MAG, MOG, and PLLP were significantly affected by rs1990622 genotype in our proteomic dataset at P < 0.05, but none remained significant after correcting for false discovery rate. Similarly, the area and intensity of MBP staining across the CA1 region of the hippocampus did not differ between rs1990622-G/G genotype and rs1990622-A allele carriers aged 78–100 (Fig. 8C, D). Rs1990622 therefore appears to have a greater impact on myelin lipids than myelin proteins.

## Discussion

The risk of dementia doubles every five years after age 65 [1], thought to result from age-dependent changes that sensitize the brain to neurodegeneration. Employing a large set of post-mortem samples, we have identified proteins whose abundance is significantly affected by physiological ageing in the hippocampus of humans aged 65 and older. Ribosomal proteins, respiratory complex proteins, and proteins involved in amino acid and lipid metabolism were increased with age, while half of the significantly decreased proteins were regulators of the cytoskeleton and cell adhesion. Lysosomal protein TMEM106B was increased with the greatest effect size, and this was specific to carriers of the rs1990622-A dementia risk allele. Accordingly, levels of sarkosyl-insoluble, fibrillar TMEM106B were significantly higher in rs1990622-A allele carriers. While *APOE* genotype had no significant effect on hippocampal lipids in our dataset, rs1990622-A was associated with lower myelin sphingolipid and higher polyunsaturated phospholipid content in carriers of the dementia risk-neutral *APOE* ε3/ε3 genotype, providing the first direct evidence for an effect of this dementia risk allele on brain lipids. Increased fibrillar TMEM106B levels with ageing and/or altered myelin lipid homeostasis are likely contributors to the increased susceptibility of rs1990622-A allele carriers to neurodegeneration.

The majority of proteins whose levels were significantly correlated with age were involved in constitutive metabolic and cellular processes such as lipid and amino acid metabolism, translation, and control of cell adhesion and the cytoskeleton. Most of the correlations between age and proteins involved in lipid metabolism (KBTBD11, METTL7A, ASAH1, ACOT7) or amino acid metabolism (PRODH, GSTM1, MARCOD1, MAOB) could be validated in either of two independent proteomic datasets from prefrontal cortex [18, 37]. The age-dependent increase in the lysosomal proteins TMEM106B and ASAH1 was validated in both independent datasets. These findings concur with increased lifetimes for lysosomal proteins in old compared to young adult mice [10].

Our observation of a general increase in electron transport chain proteins with age is in agreement with published studies on the rat hippocampus [16], but contrasts with other studies reporting either no significant change in mitochondrial proteins [18] or decreased levels of electron transport chain proteins in the hippocampus of humans aged > 90 years, compared to those aged 20 to 49 years [14, 17]. Similarly, while our study demonstrated an overall increase in abundance for ribosomal protein subunits with increasing age, prior studies have reported dissonant findings regarding changes to ribosomal protein abundance across humans, mice, monkeys, and fish [11, 15, 18, 68]. These differences are most likely attributed to experimental design. Rather than comparing mean protein levels between young and old age groups, as has been done in prior studies, we used a large sample size of individuals over the age of 65 and identified proteins whose abundance correlated with age. This allowed us to identify age-dependent changes to the proteome across the age range that is most relevant to the onset of dementia. We also note that sample sizes in prior studies with human hippocampus were limited to 3 or 4 samples per group [14, 17]. A limitation of our study is that overrepresentation of ribosomal and electron transport chain subunits may reflect the natural bias of proteomics towards more abundant proteins. Nonetheless, our findings are supported by the substantial overlap between our proteomic dataset and published gene expression data on human frontal cortex [13], in terms of proteins that increased or decreased with age (Fig. 1D).

Since our study investigated cognitively normal donors, we cannot rule out the possibility that increased levels of the lysosomal proteins TMEM106B and ASAH1 with ageing are representative of a protective response that averts neurodegeneration. In this regard, an important question is whether higher TMEM106B with ageing sensitizes to, or protects against, dementia. The former seems more likely, given that increased TMEM106B with ageing was seen only in carriers of the rs1990622-A dementia risk allele. Rs1990622-A is relatively unique among genetic risk factors for dementia, as it increases risk for all major forms of dementia [43]. This SNP is also associated with lower cognitive scores in adults without neurodegenerative diseases [69, 70] and a transcriptomic signature that characterizes accelerated ageing in people > 65 years of age [70]. Further supporting a pathogenic role for high TMEM106B protein load, our study has established that sarkosyl-insoluble, fibrillar TMEM106B is significantly higher in the hippocampus of rs1990622-A allele carriers over the age of 65, relative to those with the protective rs1990622-G/G genotype. Similarly, analysis of published proteomic data from human prefrontal cortex [37] demonstrated that levels of a peptide mapping to the C-terminal fibril-forming region of TMEM106B increased with ageing, whereas an N-terminal peptide did not. These findings suggest that the increase in TMEM106B with ageing is largely attributed to an increase in the fibrillar C-terminal fragment. In agreement with our findings, recent studies have shown age-dependent TMEM106B fibril accumulation in the frontal cortex of mixed cohorts with multiple neurodegenerative diseases [29, 51, 71]. One of these studies reported a significant association of fibrillar TMEM106B with rs1990622 genotype [51], but had too few control subjects to determine this association in subjects without dementia. Increased levels of fibrillar TMEM106B in rs1990622-A allele carriers suggest that the fibrils are neurotoxic.

Rs1990622 is in perfect linkage disequilibrium with the TMEM106B coding variant rs3173615 [49], which results in a T185S substitution within the fibril-forming C-terminal fragment. The protective Ser185 variant is associated with more rapid TMEM106B turnover in lysosomes and lower TMEM106B protein levels [72], again indicating that the dementia risk alleles are associated with higher TMEM106B. Future studies should determine whether the Thr185 variant promotes amyloidogenic TMEM106B proteolysis and/or aggregation of the C-terminal fragment. Rs1990622 is also in perfect linkage disequilibrium with the non-coding SNP rs1990620, which affects recruitment of the chromatin-organising protein CCCTC-binding factor (CTCF) to the *TMEM106B* gene locus, leading to increased *TMEM106B* gene expression [44]. This mechanism may contribute to the age-dependent increase of TMEM106B in rs1990622-A carriers, however our analysis of a large transcriptomic study of prefrontal cortex samples [38] indicated that the trend for increased *TMEM106B* mRNA with ageing was only apparent in those aged > 60, and modest in magnitude compared to the age-dependent increase in TMEM106B protein levels in the same brain region.

These SNPs in the *TMEM106B* gene increase FTD risk and decrease the age of onset in people with heterozygous *GRN* loss of function mutations [43, 73], and while the evidence presented above suggests that higher TMEM106B sensitizes to dementia with ageing, reducing TMEM106B does not correct abnormal lysosomal enzyme activity and behavioural deficits in *GRN*^*+/−*^ mice [74]. Instead, loss of TMEM106B in mice causes accumulation of vacuolar lysosomes in motor neurons, gliosis, Purkinje neuron loss, and motor deficits [75, 76]. On a *GRN*^*−/−*^ background, TMEM106B deficiency causes more pronounced lysosome abnormalities, lipofuscin accumulation, gliosis, TDP-43 pathology, motor neuron loss, and myelin loss [52, 53, 75]. Since overexpression of TMEM106B also produces adverse effects on lysosome size, acidification, and positioning [44–46], balanced TMEM106B levels are critical for correct lysosomal function and healthy brain ageing. It is possible that proteolytic formation of the C-terminal, fibrillar TMEM106B fragment results in depletion of the full-length, functional protein. We were unable to test this in our study as neither of the two antibodies that we tested produced a clear band at the correct size for TMEM106B in the sarkosyl-soluble protein fraction. Vicente et al. [51] reported a weak inverse correlation between full-length, detergent-soluble TMEM106B and the sarkosyl-insoluble fibrillar form. However, Chen-Plotkin et al. [46] reported increased levels of full-length (43 kDa) TMEM106B in FTD cases with *GRN* gene mutations, which are characterized by high TMEM106B fibril content [51].

After adjusting for the effect of age on protein levels, TMEM106B abundance was correlated with lipid metabolic and myelination protein networks that are upregulated in response to demyelination, AD, and brain ageing [63, 77]. QKI, which was correlated with TMEM106B at Q < 0.05, is a master regulator of oligodendrocyte fatty acid metabolism and myelin maintenance [58]. This protein is also necessary for the microglial phagocytosis of myelin debris [78], which may explain its association with both brain ageing and incipient AD (Fig. 3C). TMEM106B-associated protein PLXNB1 is also associated with brain ageing and severity of AD pathology [79] and has been proposed as a key driver of AD in the ageing brain [80]. We were unable to validate these correlations in two independent human prefrontal cortex datasets, potentially due to the smaller sample size for people over age 60 in these published datasets (which greatly reduces statistical power) and the fact that these studies used frontal cortex rather than hippocampus. Overall, our data suggests that TMEM106B is part of a network of proteins whose levels are increased in the ageing brain and incipient AD.

Higher myelin sphingolipid (particularly ST and Hex2Cer) content in rs1990622-G/G individuals could result from higher overall myelin content, or a more specific effect of rs1990622 on lipid metabolism or transport. The latter is supported by the observation that rs1990622 significantly affected the hippocampal lipidome but not the proteome, with little impact on myelin protein levels. Myelin is also enriched in PEp [67], and rs1990622-G/G was associated with higher levels of saturated, and lower levels of polyunsaturated, PEp. As a stable membrane with low fluidity, myelin contains more saturated/monounsaturated and less polyunsaturated lipids than other cell membranes [67]. In contrast, vesicular membranes often contain more polyunsaturated phospholipids that facilitate membrane fusion [81]. The effect of rs1990622 on hippocampal lipids may be distinct from TMEM106B protein levels since we observed no correlation between TMEM106B levels and myelin sphingolipids. However, the expression of genes involved in galactosylceramide biosynthesis is decreased in TMEM106B knockout mice [42], suggesting that TMEM106B regulates sphingolipid metabolism in oligodendrocytes, the only cells in the nervous system that synthesize galactosphingolipids [67]. Overall, an effect of rs1990622 on myelin content would be consistent with the requirement for TMEM106B in myelination [40–42]. Rs1990622 did not affect myelin content visualized by MBP staining, however definitive assessment of the effect of rs1990622 on myelin integrity requires an examination of myelin ultrastructure using electron microscopy.

Although the large sample size of neurologically normal brain donors empowered us to probe the effects of age, *APOE*, and *TMEM106B* genotype on hippocampal protein and lipid composition in individuals aged over 65, our study was limited to nine individuals with both *APOE* ɛ3/ɛ3 and rs1990622-G/G genotype. Our demonstration that rs1990622 genotype affects lipid metabolism should therefore be validated in another sample cohort, ideally sampling a white matter region given the impact of rs1990622 on myelin lipids. Rs1990622-A interacts with *APOE* genotype to increase risk for AD in the Han Chinese population [82]. The current study was underpowered to test the combined effects of *APOE* genotype and rs1990622 on the hippocampal lipidome. Given (i) that rs1990622-A increases the risk for FTD, particularly in people with heterozygous *GRN* mutations [73], and (ii) these *GRN* mutations are associated with substantially lower myelin sphingolipid content in people with FTD [39], it would also be of interest to determine if these rare *GRN* mutations are also associated with lower myelin sphingolipid content in cognitively normal human donors, and how this is affected by rs1990622-A.

## Conclusions

In summary, this study establishes that TMEM106B protein levels increase with age over 65, as part of a network of proteins that increase with age in the human hippocampus. Increased hippocampal TMEM106B with ageing is driven by the rs1990622-A dementia risk allele in the *TMEM106B* gene, which was associated with increased levels of sarkosyl-insoluble, fibrillar TMEM106B. We provide the first experimental evidence that rs1990622-A regulates brain lipid metabolism, placing it among the growing list of genetic risk factors for dementia involved in lipid homeostasis. Lastly, the matched proteomic and lipidomic datasets generated in this project should prove a valuable resource for neuroscience, ageing, and dementia research.

### Electronic supplementary material

Below is the link to the electronic supplementary material.

Supplementary Material 1

Supplementary Material 2

## Data Availability

The proteomic and lipidomic datasets supporting the conclusions of this article are provided in Additional File 1. The raw proteomic data is deposited to the ProteomeXchange Consortium via the PRIDE partner repository [83] with the dataset identifier PXD043880, and lipidomic data with Synapse, Project SynID: syn52087674 (10.7303/syn52087674).

## Abbreviations

AD: Alzheimer’s disease
ADRC: Alzheimer’s Disease Research Center
ASAH1: Acid ceramidase 1
BUME: Butanol-methanol
Cer: Ceramide
CTCF: CCCTC-binding factor
CLDN11: Claudin-11
CNP: 2’,3’-Cyclic nucleotide 3’-phosphodiesterase
DHRS7: Dehydrogenase/reductase 7
ERLIN2: ER lipid raft associated 2
FDR: False discovery rate
FTD: Frontotemporal dementia
GIS: Global internal standards
GSEA: Gene set enrichment analysis
HexCer: Hexosylceramide
Hex2Cer: Dihexosylceramide
HS-ageing: Hippocampal sclerosis with ageing
LATE: Limbic-predominant age-related TDP-43 encephalopathy
LC-MS/MS: Liquid chromatography-tandem mass spectrometry
LMNA: Lamin A/C
MAG: Myelin-associated glycoprotein
MBP: Myelin basic protein
MOG: Myelin-oligodendrocyte glycoprotein
NFT: Neurofibrillary tangle
NICHD: National Institute of Child Health and Human Development
NIMH: National Institute of Mental Health
OMG: Oligodendrocyte-myelin glycoprotein
PBS: Phosphate buffered saline
PE: Phosphatidylethanolamine
PEp: Phosphatidylethanolamine plasmalogen
PLXNB1: Plexin B1
PLLP: Plasmolipin
PLP1: proteolipid protein 1
QKI: Protein quaking I
SM: Sphingomyelin
SNP: Single-nucleotide polymorphism
ST: Sulfatide
TCEP: tris(2-carboxyethyl)phosphine
TDP-43: TAR DNA binding protein 43
TG: Triglyceride
TMEM106B: Transmembrane protein 106B
