# Correction: The major TMEM106B dementia risk allele affects TMEM106B protein levels, fibril formation, and myelin lipid homeostasis in the ageing human hippocampus

**DOI:** 10.1186/s13024-024-00709-9

**Published:** 2024-02-27

**Authors:** Jun Yup Lee, Dylan J. Harney, Jonathan D. Teo, John B. Kwok, Greg T. Sutherland, Mark Larance, Anthony S. Don

**Affiliations:** 1 Charles Perkins Centre, 2006 Camperdown, NSW Australia; 2 School of Medical Sciences, 2006 Camperdown, NSW Australia; 3 Brain and Mind Centre, The University of Sydney, 2006 Camperdown, NSW Australia

Molecular Neurodegeneration (2023) 18:63

10.1186/s13024-023-00650-3

The original article contains an error in Fig. 1C– the red label should instead indicate “Increased protein sets with ageing” and blue should instead indicate "Decreased protein sets with ageing".

